# *Carlina acaulis* Exhibits Antioxidant Activity and Counteracts Aβ Toxicity in *Caenorhabditis elegans*

**DOI:** 10.3390/molecules21070871

**Published:** 2016-07-02

**Authors:** Pille Link, Kevin Roth, Frank Sporer, Michael Wink

**Affiliations:** 1 Institute of Pharmacy and Molecular Biology, Heidelberg University, Heidelberg 69120, Germany; k.roth@dundee.ac.uk (K.R.); frank.sporer@gmx.de (F.S.); 2 Jacqui Wood Cancer Centre, Division of Cancer Research, School of Medicine, University of Dundee, Dundee, Scotland DD1 9SY, UK

**Keywords:** *Carlina acaulis*, Carlina oxide, antioxidant, beta-amyloid, *Caenorhabditis elegans*

## Abstract

*Carlina acaulis* is a medicinal plant that has shown antioxidant activity in in vitro studies, but to date no corresponding in vivo data is available. Therefore, in the present study the antioxidant activity and its impact in counteracting Aβ toxicity were studied in the *Caenorhabditis elegans* model. A dichloromethane extract of the roots of *C. acaulis* was prepared and characterised via gas-liquid-chromatography/mass-spectrometry (GLC-MS). The in vitro antioxidant activity was confirmed via 2,2-diphenyl-1-picrylhydracyl assay. The extract was further separated by thin layer chromatography into two fractions, one of which was a fraction of the dichloromethane extract of *C. acaulis* containing mostly Carlina oxide (CarOx). Different strains of *C. elegans* were employed to study the expression of *hsp-16.2p::GFP* as a marker for oxidative stress, delocalisation of the transcription factor DAF-16 as a possible mechanism of antioxidant activity, the effect of the drug under lethal oxidative stress, and the effect against beta-amyloid (Aβ) toxicity in a paralysis assay. The *C. acaulis* extract and CarOx showed high antioxidant activity (stress reduction by 47% and 64%, respectively) in *C. elegans* and could activate the transcription factor DAF-16 which directs the expression of anti-stress genes. In paralysis assay, only the total extract was significantly active, delaying paralysis by 1.6 h. In conclusion, in vivo antioxidant activity was shown for *C. acaulis* for the first time in the *C. elegans* model. The active antioxidant compound is Carlina oxide. This activity, however, is not sufficient to counteract Aβ toxicity. Other mechanisms and possibly other active compounds are involved in this effect.

## 1. Introduction

*Carlina acaulis* L. (Asteraceae) is a medicinal plant native to Central and Southern Europe. Traditionally, the root of this plant is used as diuretic and diaphoretic [1], vinegar decocts are employed to treat wounds and skin disorders [2]. The root contains 1%–2% essential oil with Carlina oxide as the main compound, comprising over 95% of the oil [3]. The essential oil, different extracts, and Carlina oxide exhibit antimicrobial activity against Gram-positive bacteria, especially *Staphylococcus aureus* [2,4,5]. Additionally, anti-inflammatory, anti-ulcer [6], and anti-trypanosomal properties [5] have been reported.

The antioxidant activity of methanol extracts of *C. acaulis* has been studied in in vitro assays, and this effect was suggested to be responsible for the anti-inflammatory and gastroprotective properties of this plant [6]. Apparently, Carlina oxide is the active antioxidant compound in the essential oil of *C. acanthifolia*, a species with a similar secondary metabolite profile as *C. acaulis* [7]. However, the antioxidant effects were only tested in vitro, to date no in vivo data is available. Therefore, in the present paper antioxidant effects were studied in vivo in a simple model organism, *Caenorhabditis elegans*, which has become an interesting system for pharmacological research [8,9,10].

Oxidative stress may be responsible for ageing and is a common characteristic of many neurodegenerative and cardiovascular diseases. In Alzheimer’s disease, the peptide beta-amyloid (Aβ) can increase the production of reactive oxygen species by interacting with transition metal ions and impairing mitochondrial function [11,12,13]. Hence, inducing oxidative stress is an important mechanism of Aβ toxicity, and antioxidants could be used to protect patients from this effect.

The aim of the present study was to prove the antioxidant activity of *C. acaulis* root in vivo and to study the contribution of this effect in counteracting Aβ toxicity. Furthermore, the active antioxidant ingredient of the root extract was identified. Induction of heat shock protein (HSP) expression, a marker for oxidative stress, was quantified in a *C. elegans* strain expressing GFP under the control of HSP promoter. Additionally, the effects on survival of wildtype worms under lethal oxidative stress were studied. Another transgenic strain expressing the human Aβ_1–42_ in muscle cells was deployed to assess the effect on Aβ toxicity in a paralysis assay. Insights into the mechanism of action were attained by studying the activation of the transcription factor DAF-16, which regulates the expression of stress genes, in a strain expressing *DAF-16::GFP*.

## 2. Results and Discussion

### 2.1. Characterisation of the Extract and Isolation of Carlina Oxide

**Figure 1:**
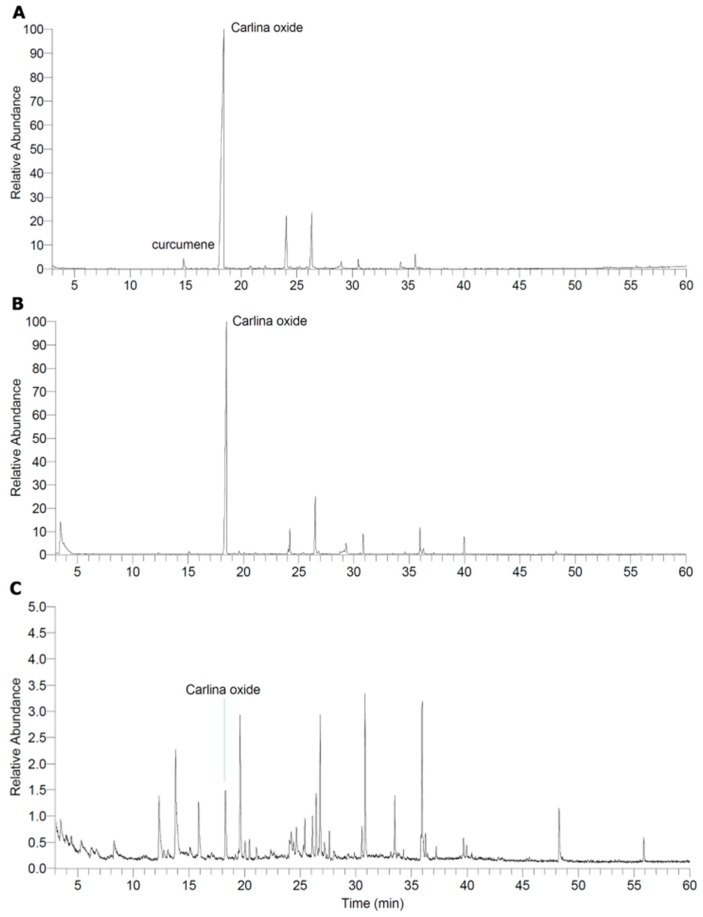
Total ion current of the GLC-MS measurements for the total dichloromethane extract of *C. acaulis* (**A**); fraction of the dichloromethane extract of *C. acaulis* containing mostly Carlina oxide (CarOx) (**B**); and the depleted extract (**C**).

The dichloromethane extract of *C. acaulis* was analysed by gas-liquid-chromatography/mass-spectrometry (GLC-MS) (Figure 1A). It contains high amounts of Carlina oxide (45% of all detected compounds based on peak area) and small amounts of curcumene (1%) that has been reported in the essential root oil of *C. acaulis* before [3]. Separation of the extract by preparative thin layer chromatography (pTLC) resulted in two fractions: CarOx, which contains 99% of the recovered Carlina oxide, and a fraction that is essentially free of this compound (depleted extract) (Figure 1B,C). CarOx is comprised of 53% Carlina oxide, and the relative amount of all other compounds was below 10%; therefore, the activity of this fraction in the following experiments was considered representative for pure isolated Carlina oxide.

### 2.2. Antioxidant Activity In Vitro

In vitro antioxidant activity of the total dichloromethane extract of *C. acaulis* was tested in the 2,2-diphenyl-1-picrylhydracyl (DPPH^●^) assay, resulting in a half maximal effective concentration (EC_50_) of 122 μg/mL. In the study of Đorđević, Tadić, Petrović, Kukić-Marković, Dobrić, Milenković, and Hadžifejzović [6], an EC_50_ of 208 μg/mL has been reported for a methanol extract of *C. acaulis* roots in the same assay, and the essential oil of *C. acanthifolia* had an EC_50_ of 13.6 μg/mL [7]. The value for the dichloromethane extract lies between those two, suggesting that the active antioxidant compound is lipophilic, mostly present in the essential oil and to a lesser amount in the respective extracts. This corresponds well with the report that Carlina oxide is responsible for the radical scavenging activity [7].

### 2.3. Antioxidant Activity in *C. elegans*

**Figure 2:**
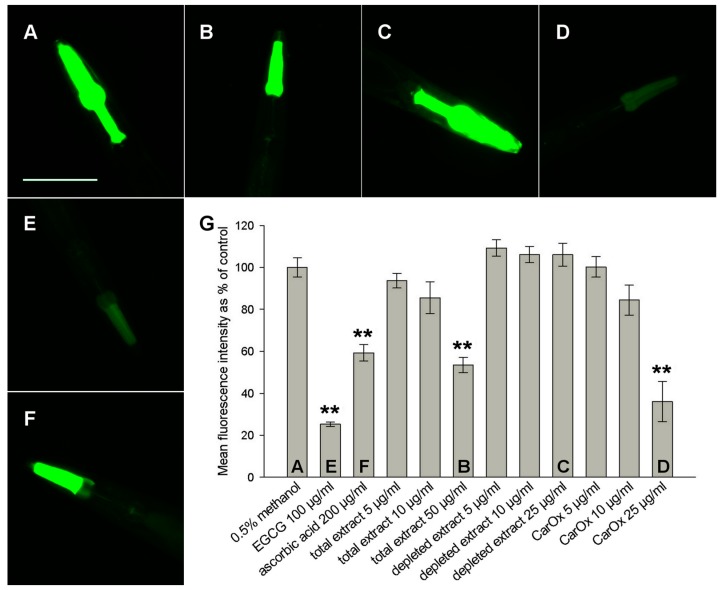
Heat shock protein (HSP) expression assay in *C. elegans*. A–F: representative pictures of GFP fluorescence in worms treated with 0.5% methanol (**A**; negative control); 50 μg/mL total extract of *C. acaulis* (**B**); 25 μg/mL depleted extract (**C**); 25 μg/mL CarOx (**D**); 100 μg/mL (−)-epigallocatechin gallate (EGCG) (**E**; positive control); and 200 μg/mL ascorbic acid (**F**; positive control); (**G**) quantification of the fluorescence intensity for respective treatments. Pictures taken with BZ9000 from Keyence, scale bar = 100 μm. ** *p* < 0.01 compared to the negative control.

In vivo antioxidant activity was tested in *C. elegans*. Juglone, a pro-oxidant naphthoquinone from *Juglans regia*, was used to induce oxidative stress in this model and the expression of GFP under the control of a HSP promoter was evaluated as an indicator of stress levels. Figure 2 shows representative fluorescence images (A–F) and the quantification of fluorescence intensity after different treatments (G). The positive control (−)-epigallocatechin gallate (EGCG) lowered the stress level by 75%, a value similar to the 73% decrease reported by Abbas and Wink [14]. The total extract of *C. acaulis* and CarOx showed dose-dependent activity with the highest tested concentrations reaching significant decrease in stress level (47% for 50 μg/mL total extract, 64% for 25 μg/mL CarOx, *p* < 0.0001), being more effective than ascorbic acid (41% decrease, *p* < 0.0001). The depleted extract did not show significant activity. These results further support the assumption that Carlina oxide is the active antioxidant compound in *C. acaulis* roots. Furthermore, its in vivo activity was shown for the first time.

**Figure 3:**
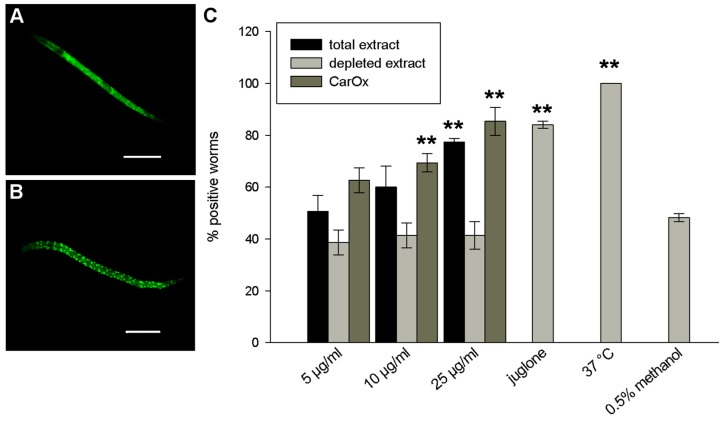
Results of the DAF-16 delocalisation assay in *C. elegans*. (**A**) A worm treated with 0.5% methanol (negative control); (**B**) a worm treated with 25 μg/mL CarOx. Pictures taken with BZ9000 from Keyence, scale bars = 100 μm; (**C**) quantification of the results. ** *p* < 0.01 compared to the negative control.

In order to find out whether the antioxidant activity is based solely on the radical scavenging capacity of Carlina oxide or whether other mechanisms are involved, the DAF-16 delocalisation assay was employed. DAF-16, the *C. elegans* homologue of human FOXO, is a transcription factor that is activated under stress and initiates the expression of different protective genes, including antioxidant proteins like superoxide dismutase or glutathione S-transferase [15,16]. Both the total extract of *C. acaulis* and CarOx induced the delocalisation of DAF-16 into the nucleus, in contrast to the depleted extract that showed no effect (Figure 3). The activity of the extract and CarOx was dose-dependent, with CarOx reaching a significant increase in nuclear localisation at a concentration of 10 μg/mL (69% and 85% of worms were positive for 10 and 25 μg/mL, respectively, *p* < 0.0001) and the total extract at 25 μg/mL (77% of worms positive, *p* < 0.0001). Hence, activation of innate protective mechanisms also plays a role in the in vivo antioxidant activity of *C. acaulis*.

**Figure 4:**
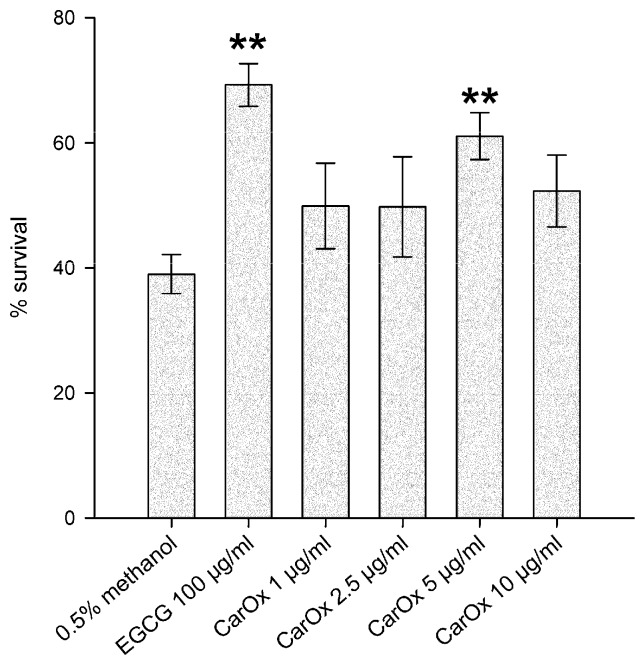
Survival assay in *C. elegans*. The worms were treated with indicated substances prior to subjecting them to lethal oxidative stress induced by 80 μM juglone. ** *p* < 0.01 compared to the negative control (methanol).

To further investigate the antioxidant properties of CarOx, the survival assay was deployed. In this assay, the worms, treated with the substance of interest, are exposed to lethal oxidative stress through a high concentration of juglone. Since Carlina oxide is not very stable, a new extract was prepared and CarOx isolated as before. It was tested in a concentration range 1–10 μg/mL. Lower concentrations as in former experiments were chosen because of observed toxicity for 25 μg/mL for the fresh CarOx. As can be seen on Figure 4, the positive control EGCG led to a significant increase in survival rate under oxidative stress (69.2% survivors, *p* = 0.0018). A treatment with 5 μg/mL CarOx also led to a significant increase in the survival (61.1% survivors, *p* = 0.0093). This result further confirms the antioxidant activity of CarOx.

It is noteworthy that the freshly prepared CarOx exhibited higher toxicity and a narrow activity range compared to the first batch of the substance. It is known that Carlina oxide has antimicrobial and antitrypanosomal activity [5]; therefore, it is not very surprising that it is also toxic to nematodes like *C. elegans*. However, the shift towards higher toxicity suggests that there are major differences between the two batches of CarOx. Carlina oxide is not a stable molecule—left at room temperature it degrades within days [17]. Hence, it is possible that a slight degradation of Carlina oxide in the first batch led to decreased toxicity. On the other hand, the first batch of CarOx still showed dose-dependent activity at non-toxic concentrations, implying that there are other active compounds in the CarOx fraction—possibly the degradation products. This assumption needs further investigation.

### 2.4. Effect Against Aβ Toxicity

**Figure 5:**
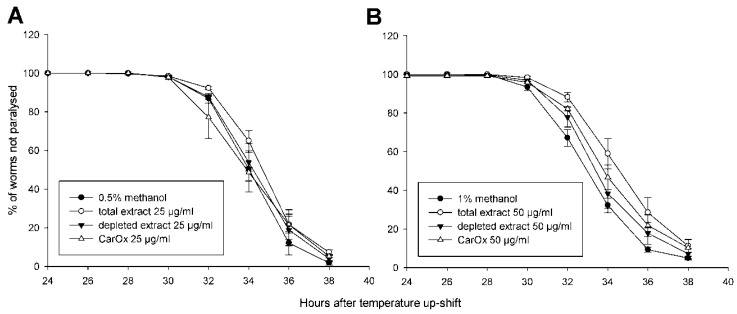
Paralysis curves for *C. elegans* expressing Aβ (CL4176) treated with dichloromethane extract of *C. acaulis* (Carlina), depleted extract, or CarOx in concentrations 25 μg/mL (**A**) and 50 μg/mL (**B**) with respective solvent controls.

Since a notable antioxidant activity could be seen in *C. elegans*, it was further investigated if this effect helps to counteract Aβ toxicity in worms expressing human Aβ peptide. In the strain CL4176, the Aβ peptides are expressed and aggregate in the muscle cells, leading to oxidative stress and eventually paralysis of the worms [18]. The results of a paralysis assay after treatment with the total extract of *C. acaulis*, depleted extract, and CarOx are shown in Figure 5 and Table 1. The control strain CL802 showed no paralysis regardless of treatment (data not shown). In CL4176, the median paralysis time (PT_50_) was not significantly affected by any of the treatments at a concentration of 25 μg/mL. When the concentration was raised to 50 μg/mL, the total extract significantly delayed paralysis, but neither CarOx nor depleted extract had a comparable effect. Hence, the effect seen in this assay for the extract cannot be attributed to Carlina oxide alone.

The results of the paralysis assay indicate that the protective effect against Aβ toxicity cannot be attributed to the antioxidant activity of *C. acaulis*. In both, the HSP-expression and DAF-16 delocalisation assays CarOx had a stronger effect than the total extract in contrast to a constant lack of activity for the depleted extract. In the paralysis assay, both CarOx and depleted extract show a small, insignificant effect, in contrast to the stronger activity of the total extract. Additive or synergistic effects of the constituents in CarOx and depleted extract seem to be necessary for significant protection against Aβ, while Carlina oxide alone is able to produce the antioxidant effect.

The activation of DAF-16 triggers the expression of antioxidant proteins but can also protect the organism from other kinds of stress. It has been shown that DAF-16 activity is also involved in Aβ detoxification [19]. However, the results of the present study suggest that DAF-16 activity alone is not sufficient to protect *C. elegans* from Aβ toxicity, since CarOx could activate this transcription factor but was not effective in the paralysis assay. Cohen, Bieschke, Perciavalle, Kelly, and Dillin [19] also reported HSF-1, another transcription factor in *C. elegans*, to be involved in the detoxification via a different pathway. The activity of both of these transcription factors might be needed to successfully counteract Aβ toxicity.

## 3. Materials and Methods

### 3.1. Chemicals and Reagents

Juglone (5-hydroxy-1,4-naphthalenedione), EGCG, and DPPH^●^ were purchased from Sigma-Aldrich GmbH (Darmstadt, Germany), and sodium azide and ascorbic acid were purchased from AppliChem GmbH (Darmstadt, Germany).

### 3.2. Plant Material

Dried and pulverised roots of *Carlina acaulis* were purchased from Caesar & Loretz GmbH (Lot 81805079); a voucher specimen with the registration number P8082 is stored at Department of Biology, Institute of Pharmacy and Molecular Biotechnology, Heidelberg University, Heidelberg, Germany.

The root powder was exhaustively extracted with dichloromethane in a Soxhlet extractor. The solvent was evaporated and the solvent-free extract (3.7% of the drug weight) was dissolved in methanol for further experiments. Separation of the extract into CarOx and a fraction depleted of Carlina oxide was carried out by preparative thin layer chromatography (pTLC) (silica gel 60, F_254_, 0.5 mm from Merck) with 90% ethylacetate and 10% cyclohexane as mobile phase. The fractions were extracted from the silica gel with methanol. The extract and fractions were stored in the dark at −20 °C.

### 3.3. GLC-MS

The total extract and both fractions were analysed via GLC-MS using an HP5890 Series II gas chromatograph (Hewlett Packard, Palo Alto, CA, USA) equipped with an OV-5 capillary column (30 m; 0.25 mm inner diameter; 0.25 μm film thickness). The injector was operated at a 250 °C in split mode (1:50) at a head pressure of 15 kPa of helium. The temperature program started with an isothermal step at 100 °C for 2 min. The temperature was then raised to 300 °C at a rate of 3 °C/min and held for 10 min. The GLC was coupled to a quadrupole mass spectrometer SSQ 7000 (Thermo-Finnigan, Bremen, Germany). Mass spectra were recorded at 70 eV at a source temperature of 175 °C with the Xcalibur™ 1.3 software (Thermo Fischer Scientific, Bremen, Germany).

The compounds were identified by comparing their mass spectra and Kovats retention index with data present in our local database, the Chemical Abstract Service database, and the literature [20,21].

### 3.4. DPPH^●^-Assay

In vitro radical scavenging activity of the total extract of *C. acaulis* was tested using the DPPH^●^-assay [22]. A 0.2 mM solution of DPPH^●^ in methanol was used and mixed 1:1 with the tested extract concentration (10–800 μg/mL in methanol). The absorbance was detected after 30 min incubation at room temperature at 517 nm (Asys UVM 340 microplate reader, anthos Mikrosysteme GmbH, Krefeld, Germany). The absorbance of the respective extract concentrations without DPPH^●^ was measured in the same manner and subtracted from the DPPH^●^ absorbance values.

### 3.5. *Caenorhabditis Elegans* Strains and Culture Conditions

All strains used in this study and *Escherichia coli* OP50 (as a food source) were obtained from the *Caenorhabditis* Genetics Center at the University of Minnesota, USA. The strains N2 (wildtype), TJ375 [*hsp-16.2p::GFP*], and TJ356 [*daf-16p::daf-16a/b*::GFP + *rol-6*(su1006)] were maintained at 20 °C, the temperature sensitive strains CL4176 [*smg-1*(cc546); *myo-3p::Aβ_1–42_*::let-3′UTR + *rol-6*(su1006)] and CL802 [*smg-1*(cc546); *rol-6*(su1006)] at 16 °C. Nematode growth medium plates and liquid S-medium with *E. coli* OP50 were used as described previously [14], and age-synchronised worms for the experiments were obtained by hypochlorite treatment [23].

### 3.6. HSP-Expression Assay

The expression of *hsp-16.2p::GFP* construct was quantified in the strain TJ375 as described previously [14]. The worms were treated with 5, 10, or 25 μg/mL of CarOx or depleted extract; 5, 10, or 50 μg/mL of total extract of *C. acaulis*; 100 μg/mL EGCG and 200 μg/mL ascorbic acid as positive controls; and the solvent methanol (0.5%) as negative control. The worms were mounted on object slides in 10 mM sodium azide in PBS. Pictures were taken of 25 worms per sample under fluorescence microscope BZ9000 (Keyence, Osaka, Japan; GFP filter, 20× objective, exposure time 1/11 s) and analysed with ImageJ 1.43u (Rasband, W.S., U. S. National Institutes of Health, Bethesda, MD, USA, http://imagej.nih.gov/ij/).

### 3.7. DAF-16 Delocalisation

The total extract of *C. acaulis*, CarOx, and depleted extract (in concentrations of 5, 10, or 25 μg/mL) were studied for their ability to delocalise the transcription factor DAF-16 into cell nuclei in the *C. elegans* strain TJ356 [15]. The worms were treated on Day 1 after hatching and evaluated 1 h later under fluorescence microscope BZ9000 from Keyence. As positive controls, 20 μM juglone and heat shock at 37 °C for 15 min were used, and the negative control was treated with the solvent methanol (0.5%). For each sample, 25 worms were evaluated. Worms with fluorescence in nuclei were scored as positive, and worms with uniform fluorescence scored as negative. The percentage of positive worms was calculated for each sample.

### 3.8. Survival Assay

The survival assay was conducted as described previously [14]. Briefly, N2 worms were treated with CarOx in concentrations of 1, 2.5, 5, or 10 μg/mL. A portion of 100 μg/mL EGCG was used as positive control and the solvent methanol (0.5%) as negative control. A portion of 80 μM juglone was used to induce lethal oxidative stress, and the survivors were counted 24 h after this induction.

### 3.9. Paralysis Assay

The effects of total extract of *C. acaulis*, CarOx, and depleted extract (in concentrations of 25 or 50 μg/mL) against Aβ toxicity were tested in the *C. elegans* strain CL4176 with temperature inducible expression of human Aβ_1–42_ as described earlier [24]. The negative control was treated with the respective amount of methanol. The strain CL802 was used as a control for Aβ-independent effects. Age-synchronised populations were generated by hypochlorite treatment, and about 100 worms per plate were used. Paralysis was scored 24 h after temperature up-shift every 2 h, up to 14 h in total.

### 3.10. Statistical Analysis

All experiments were repeated three times (*n* = 3), the results are expressed as mean ± SEM. The EC_50_ of the DPPH^●^-assay was calculated with SigmaPlot 11.0 (Systat Software, Chicago, IL, USA). following the guidelines published by Sebaugh [25]. The results of HSP-expression, DAF-16 delocalisation, and survival assays were compared using one-way analysis of variance (ANOVA) followed by Dunnett’s post-hoc test with the significance level 0.05. The PT_50_’s for the paralysis assay were calculated in a survival analysis using the life table method, and the differences were compared as in former assays.

## 4. Conclusions

The total extract of *C. acaulis* and the Carlina oxide containing fraction CarOx showed a marked antioxidant activity in *C. elegans*. Both radical scavenging activity and activation of innate protective mechanisms play a role in this effect. Antioxidant activity and activation of transcription factors like DAF-16 can play a role in the mechanism of action against proteotoxicity induced by Aβ or other aggregating peptides. In the case of *C. acaulis*, these effects are not sufficient, as shown by the lack of activity for CarOx in the paralysis assay, although the total extract was effective. The active compounds and the mechanism of action for the total extract in counteracting Aβ toxicity need further investigation. The in vivo antioxidant effect might be, however, interesting for alleviating oxidative stress in several diseases and also in healthy ageing. Further tests in vertebrate models are needed to corroborate the efficacy and safety of the drug.

## Figures and Tables

**Table 1:** PT_50_ values in hours for the treatments in paralysis assay (Figure 5). Significance was tested against the corresponding solvent control.

Treatment	PT_50_ ± S.E.M	Significance
0.5% methanol	36.0 ± 0.1	
total extract 25 μg/mL	36.7 ± 0.3	
depleted extract 25 μg/mL	36.3 ± 0.4	
CarOx 25 μg/mL	35.9 ± 0.6	
1% methanol	35.0 ± 0.2	
total extract 50 μg/mL	36.6 ± 0.5	*p* = 0.02
depleted extract 50 μg/mL	35.5 ± 0.4	
CarOx 50 μg/mL	36.0 ± 0.4	

## Abbreviations

The following abbreviations are used in this manuscript:

Aβ	beta-amyloid
CarOx	fraction of the dichloromethane extract of *C. acaulis* containing mostly Carlina oxide
DPPH^●^	2,2-diphenyl-1-picrylhydracyl
EGCG	(−)-epigallocatechin gallate
EC_50_	half maximal effective concentration
GLC-MS	gas-liquid-chromatography/mass-spectrometry
HSP	heat shock protein
PT_50_	median paralysis time
pTLC	preparative thin layer chromatography
